# In response to letter to the editor: calcar fracture gapping: a reliable predictor of anteromedial cortical support failure after cephalomedullary nailing for pertrochanteric femur fractures

**DOI:** 10.1186/s12891-022-05689-9

**Published:** 2022-07-28

**Authors:** Shi-Min Chang, Wei Mao, Shi-Jie Li, Hui Song

**Affiliations:** Department of Orthopaedic Surgery, Yangpu Hospital, Tongji University School of Medicine, Shanghai, China

**Keywords:** Trochanteric hip fracture, Cephalomedullary nail, Anteromedial cortical support, Fracture gap, Fluoroscopy

## Abstract

We appreciate the interest by Drs. Hagiyama and coauthors in our work entitled “Calcar fracture gapping: a reliable predictor of anteromedial cortical support failure after cephalomedullary nailing for pertrochanteric femur fractures”. They discussed several pertinent points and it is our pleasure to respond their concerns in order. Firstly, we agree that calcar fracture gap and anteromedial cortical support are different concepts, though both of them were used to evaluate the displacement of fracture reduction quality. Secondly, our primary outcome parameter was the threshold distance of calcar fracture gapping in anteroposterior and lateral fluoroscopies, which was calculated based on sensitivity and specificity by receiver operating characteristic curves. Thirdly, we took immediate post-operative fluoroscopic images in 3 views to describe the initial reduction quality as baseline to compare and calculate the changes with three-dimensional computed tomography, which was taken about one week after operation for confirming secondary stability after head-neck sliding and impaction. Lastly, the parameters selected in multivariable analysis. Future work with better study-design is needed to improve the prediction of patient outcomes.

We appreciate the interest in our work by Drs. Hagiyama and coauthors. They discussed several pertinent points [1]. Here we respond their concerns in order.

## First is about the concept of calcar fracture gap and anteromedial cortical support

**Fig. 1:**
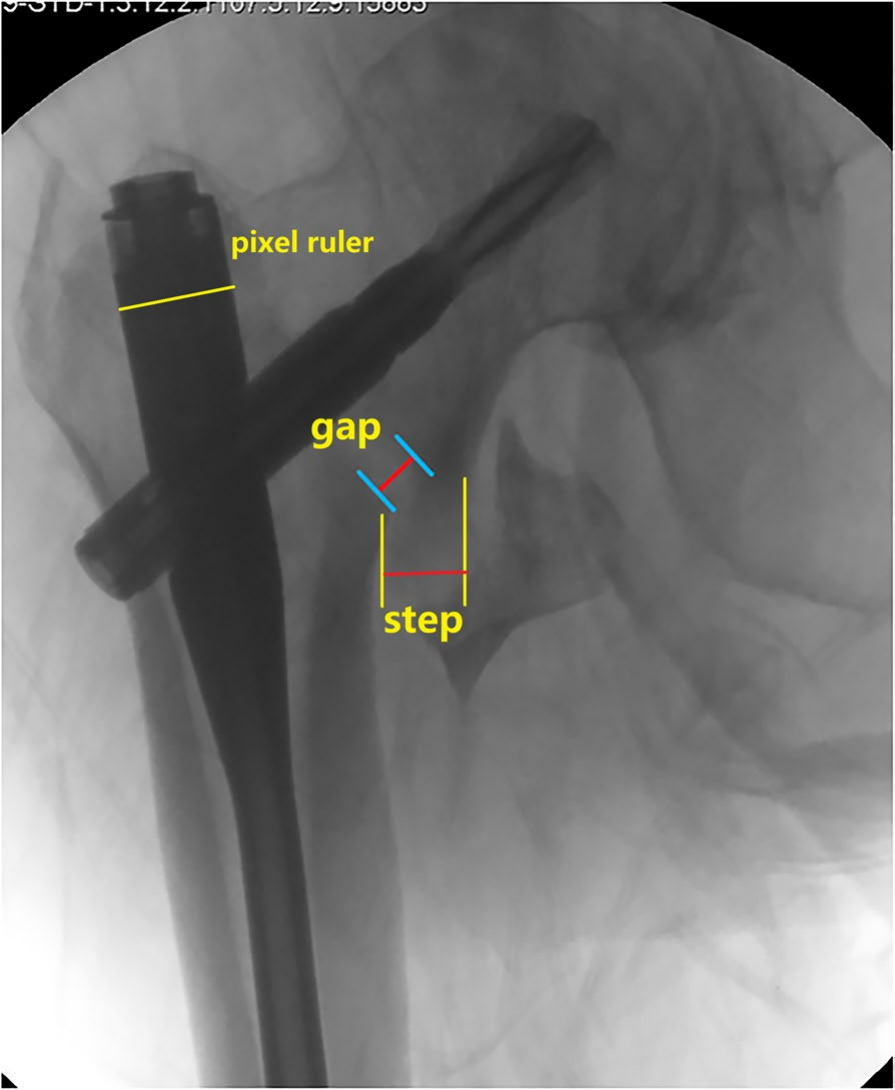
Measuring calcar fracture gap and cortical step at the anteromedial inferior corner after fracture reduction and cephalomedullary nailing, using immediate post-operative fluoroscopic image. The proximal nail diameter is used as calibrator

We agree that calcar fracture gap and anteromedial cortical support are different concepts, though both of them were used in evaluating the fracture reduction quality. For trochanteric fractures, the fracture reduction quality is evaluated by Garden alignment and fragment displacement. Usually, two indexes are applied to describe fragment displacement: gap and step (Fig. 1). Calcar fracture gap is the axial distance between the two main fragments (the head-neck and the shaft), measured from the inner cortical margin of the neck fragment, and along the implant sliding direction (usually 130 degrees in cephalomedullary nails in AP view) [2, 3]. The anteromedial cortical support emphasizes the relative position (or azimuth bearing in geometric) of the proximal head-neck fragment, which delineates the status of perpendicular cortical step-off at the anteromedial inferior corner, i.e. positive = extramedullary end-to-side cortical apposition = over-reduction, neutral = smooth end-to-end cortical apposition, which is not synonym to anatomic reduction, and negative = intramedullary cortical apposition = inadequate reduction = loss of cortical contact or no cortical support [4].

We agree to the point that measuring calcar fracture gap is meaningless in the mode of negative anteromedial cortex relation. In our study, those patients were excluded, i.e. we only included patients with non-negative cortical relations at immediate post-operative fluoroscopy. We think it is suitable to estimate the threshold values by receiver operating characteristic (ROC) curves.

## Second is the main end point

Our primary outcome parameter was the threshold distance of calcar fracture gapping in AP and lateral fluoroscopies, which was calculated based on sensitivity and specificity by ROC curves. The threshold predicted the anteromedial calcar support failure later in follow-up and demonstrated by 3D-CT images, which provided 360-degree full range views of the cortex.

## Third is the application of fluoroscopy and 3D-CT

Fracture reduction quality can be assessed at different points of time-line with different imaging modalities, usually by fluoroscopy in the operating theater (intra-operative monitoring, immediate post-operative evaluation), and by radiography, CT scanning, and 3D reconstruction post-operatively. As a routine, we take immediate post-operative fluoroscopic images in 3 views of the AP, lateral and 30-degree anteromedial oblique tangential [5], to describe the initial reduction quality (which maybe the real status with fully active compression by surgeon, or possible tendency after sliding with passive impaction), and use these images as baseline to compare and calculate the changes in follow-ups, and to find out the predictive risk factors for final reduction failure. In addition, as described in the text, our data were measured and calculated by using Photoshop CC 2018 (Adobe) software with the pixel ruler technique. We used the proximal nail diameter (16.5 mm) as the calibrator [6].

It is well known that when the operated patient is left the fracture traction table, the head-neck fragment position may accordingly change. For example, it could happen in the transport process from the operating room (with fluoroscopy) to the post-anesthesia care unit (with radiograph) [7].

We agree that 3D-CT is more accurate in assessment and measurement. However, CT has a large amount of radiation exposure and usually not available during operation for the majority of hospitals. In our practice, CT scanning and 3D reconstruction is used to confirm secondary stability after head-neck telescoping and impaction to achieving cortical contact in follow-up. Our CT examination is taken about one week after operation in the radiology department.

We are happy to read the article by Dr. Yamamoto (the second and corresponding author of the comment letter) and his colleagues, which reported that compared to plain radiographs, postoperative CT assessment of anteromedial cortex reduction is a good predictor for reoperation after intramedullary nail fixation for pertrochanteric fractures [8]. The authors classified patients into 3 different groups according to fracture reduction quality assessed by postoperative 3D-CT, and measured some variables such as sliding distance in follow-up radiographs taken postoperatively in 2 weeks and 3 months. However, throughout the paper, the authors did not mention any information about when the first postoperative CT examinations were performed. Obviously, fracture reduction pattern may be changed after movement and full weight-loading. The reduction pattern obtained on the immediate postoperative CT examinations may be different from the result obtained later, for example in 2 weeks after surgery, i.e. the later the CT examination, the closer the finding to the final.

## Fourth is the selection of parameters in multivariable analysis

We agree in general that the selection of explanatory variables should be based on the association with the objective variables, rather than solely on a *p*-value (*p* < 0.10) in univariate analysis [9]. However, as for simplicity in practice, it is acceptable indeed for small sample clinical studies [10, 11]. Stability of bone-implant after fracture fixation is related to 5 factors: bone quality, fragment geometry, fracture reduction quality, implant selection, and implant placement. Surgeons have no control over the bone quality (osteoporosis) and fragment geometry (fracture classification), and we believe there is no reason to suspect a difference in these 2 factors between our low-energy geriatric hip fracture groups. In addition, all surgeries were performed or supervised by a small group of experienced orthopedic trauma specialist. The accuracy of the tip-apex distance (TAD) and calcar-referenced TAD (Cal-TAD) seen across all groups attests to the precision of the surgery performed as well as the consistency of precision across groups. Overall, we think the risk of these confounders is low, though we would agree that there is a potential for bias related to osteoporosis and/or surgeon’s experience factors. Furthermore, as the calcar fracture gap and anteromedial cortical support are two different concepts, we insist that both these parameters should be included in multivariable analysis.

As for a tridimensional structure of the head-neck and shaft fragment, it is not surprising that fracture displacement parameters from different perspectives may interrelate with each other to some extent, so any suboptimal fracture reduction in one parameter may influence the whole position of the head-neck fragment and the final outcomes [12]. We would like to investigate these parameter relations in the future.

In summary, it is beneficial to investigate the prognostic risk factors leading to anteromedial cortical support failure for trochanteric hip fractures treated with cephalomedullary nails, at different time points with different imaging modalities. We hope that future work with better study design will help predict and improve patient outcomes. We appreciate the authors’ work in this field and their critical comments, and the opportunity for our further clarification.

## Data Availability

The datasets used and analyzed in the original study are available from the corresponding author on reasonable request.

## Abbreviations

AP: Antero-posterior
3D-CT: Three dimensional computed tomography
TAD: Tip-apex distance
Cal-TAD: Calcar-referenced tip-apex distance
